# Mucin-Phenotype and Expression of the Protein V-Set and Immunoglobulin Domain Containing 1 (VSIG1): New Insights into Gastric Carcinogenesis

**DOI:** 10.3390/ijms24108697

**Published:** 2023-05-12

**Authors:** Catalin-Bogdan Satala, Ioan Jung, Simona Gurzu

**Affiliations:** 1 Department of Pathology, George Emil Palade University of Medicine, Pharmacy, Science and Technology, 540142 Targu Mures, Romania; stlcatalin92@yahoo.com (C.-B.S.); jungjanos@studium.ro (I.J.); 2 Department of Pathology, Clinical County Emergency Hospital, 540136 Targu Mures, Romania; 3 Research Center for Oncopathology and Translational Medicine (CCOMT), George Emil Palade University of Medicine, Pharmacy, Science and Technology, 540136 Targu Mures, Romania

**Keywords:** gastric cancer, intestinal metaplasia, *MUC2*, *MUC5AC*, *CDX2*, *VSIG1*

## Abstract

In gastric cancer (GC), intestinal metaplasia (IM) is a common precursor lesion, but its relationship to the *MUC2/MUC5AC/CDX2* axis is not completely understood. Although V-set and immunoglobulin domain containing 1 (*VSIG1*) is supposed to be a specific marker for gastric mucosa and GC, respectively, no data about its relationship with IM or mucin phenotype have been published. The aim of our study was to explore the possible linkage between IM and these four molecules. The clinicopathological features of 60 randomly selected GCs were examined in association with VSIG1, MUC2, MUC5AC and CDX2. Two online database platforms were also used to establish the transcription factors (TFs) network involved in *MUC2/MUC5AC/CDX2* cascade. IM was more frequently encountered in females (11/16 cases) and in patients below 60 years old (10/16 cases). Poorly differentiated (G3) carcinomas tended to show a loss of CDX2 (27/33 cases) but not of MUC2 and MUC5AC. MUC5AC and CDX2 were lost in parallel with the depth of invasion of the pT4 stage (28/35 and 29/35 cases), while an advanced Dukes-MAC-like stage was only correlated with CDX2 and VSIG1 loss (20/37 and 30/37 cases). VSIG1 was directly correlated with MUC5AC (*p* = 0.04) as an indicator of gastric phenotype. MUC2-negative cases showed a propensity towards lymphatic invasion (37/40 cases) and distant metastases, while CDX2-negative cases tended to associate with hematogenous dissemination (30/40 cases). Regarding the molecular network, only 3 of the 19 TFs involved in this carcinogenic cascade (*SP1*, *RELA*, *NFKB1*) interacted with all targeted genes. In GC, VSIG1 can be considered an indicator of gastric phenotype carcinomas, where carcinogenesis is mainly driven by *MUC5AC*. Although infrequently encountered in GC, CDX2 positivity might indicate a locally advanced stage and risk for vascular invasion, especially in tumors developed against the background of IM. The loss of VSIG1 indicates a risk for lymph node metastases.

## 1. Introduction

In the era of personalized medicine and the targeted therapy of cancer, gastric cancer (GC) remains the fifth most frequent type of cancer and the fourth in terms of mortality rates worldwide [1]. Despite the advances that have been made in recognizing and, to some extent, treating the precursor conditions, the incidence of GC is still high, mainly due to improvements in diagnostic tools, especially in the endoscopy field.

GC is well known to be one of the most heterogeneous tumor types, both phenotypically and genetically [2,3]. Lauren’s classification, the first ever published, has had more than just a historical impact: the intestinal and diffuse subtypes are still used for diagnosis today [4]. However, the variants have been updated with improvements in the methods used to establish tumor phenotypes. The intestinal and gastric phenotypes can be determined using either immunohistochemical (IHC) markers or molecular biology techniques. 

Recently, increasing evidence has suggested that the mucin immunophenotype is not only helpful in classifying the GC but also has prognostic significance [5,6,7]. Mucins are glycoproteins secreted in a variety of tissues, both normal and neoplastic. In physiological conditions, mucins play multiple roles: they are an integral part of the mucosa protective barrier and are actively involved in chemical and molecular interactions at the mucosa–mucus interface. Recent studies emphasized the possible use of the mucus layer as a drug-delivery target due to its mucoadhesion properties [8,9,10]. 

In carcinogenesis, the role of mucins is diverted through either the glycosylation or neosynthesis of different phenotypes that are not characteristic of the tissue of tumor origin [11,12]. Mucin phenotype can be a useful tool in the identification of the preneoplastic lesion. On the one hand, atrophic gastritis is mostly associated with incomplete intestinal metaplasia (IM; type II and III), which exhibits positivity for both MUC2 and MUC5AC. On the other hand, complete IM (type I) is characterized by immunohistochemical (IHC) positivity for MUC2 only. This pathway is more frequently encountered in peptic ulcer-neighboring mucosa. It has also been shown that MUC5AC marks dysplastic mucosa, associated with either gastritis or gastric peptic disease, whereas MUC2 is expressed in mucosa with IM, irrespective of the presence or absence of dysplasia [13,14]. 

Though the involvement of mucins in GC has been intensively studied, to date, there is a lack of complete understanding of the roles of mucins, especially *MUC2* and *MUC5AC*, in the molecular carcinogenic cascade. This is primarily due to the intricate and complex mechanisms through which they interfere with other biomolecules, such as transcription factors (TFs) and microRNAs (miRNAs) that affect other major molecular pathways. 

In this study, we aimed to demonstrate the possible predictive impact of mucin phenotype in GC, as well as to elucidate, at least partially, the complex mucin molecular networks involved in gastric carcinogenesis. An additional aim of the paper was to determine how the V-set and immunoglobulin domain containing 1 (VSIG1), a recently identified protein of the gastric mucosa, with emerging potential impact in GC, is involved in mucin synthesis in gastric carcinogenesis and how this molecule influences the clinicopathological features of GC patients [15,16]. 

## 2. Results

**Clinicopathological features associated with intestinal metaplasia (IM):** In our study, most patients were men over 60 years of age, and the age range of the entire cohort was 39 to 85 years. Of the 60 patients included in this study, only 16 had tumors associated with IM as a precursor lesion. In these 16 patients, the female sex was dominant, representing 11 cases. In contrast, there was a strong negative correlation between the male sex and IM as a precursor lesion: the category without IM was almost exclusively comprising male patients, with only 2 out of 44 cases being women. IM seemed to be more frequently observed in patients under their 60: only 6 out of the 16 tumors associated with IM were in patients over 60 years, while in the category of “no associated IM”, most patients were over 60 years (Table 1).

Helicobacter pylori infection status was assessed in the peri-tumoral gastric mucosa, using immunohistochemical methods, in 42/60 cases. Fifteen out of the forty-two cases (35.71%) were associated with H. pylori infection, all of them also presenting IM.

Regarding tumor size, most tumors (38/60) showed a maximum diameter of <30 mm, without correlation with presence or absence of IM (*p* = 0.599). None of the other clinicopathological data analyzed, including angio-lymphatic or perineural invasion (*p* = 0.935), showed statistically significant correlation with IM (Table 1).

**Figure 1:**
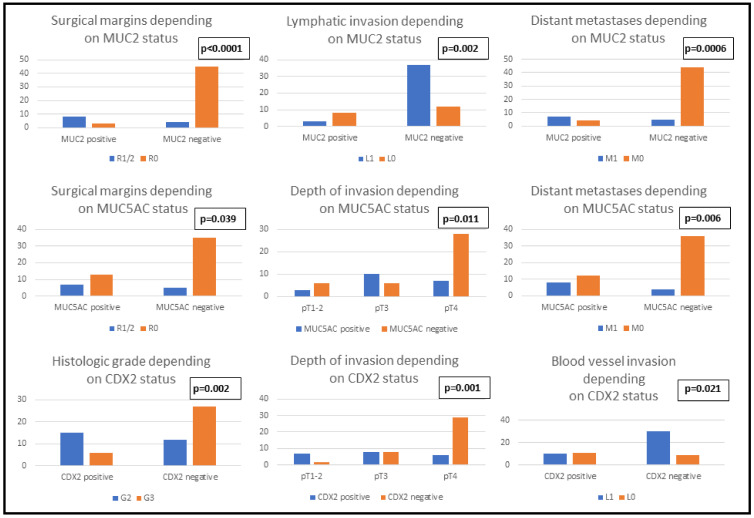
Clinicopathological parameters of patients with gastric cancer correlated with the immunophenotype of MUC2, MUC5AC and CDX2.

**Clinicopathological features and immunophenotype of MUC2, MUC5AC and CDX2:** In our cohort, the IHC positivity for MUC2 and CDX2 was associated with the female sex, independent to the age of the patients. Poorly differentiated tumors tended to exhibit CDX2 negativity independent to their size (*p* = 0.129). Regarding tumor stage, cases with advanced depth of invasion (pT4) demonstrated a tendency towards a loss of MUC5AC and CDX2 but not MUC2. On the other hand, the Dukes-MAC-like stage was correlated only with CDX2 phenotype: the loss of CDX2 expression was mainly observed in advanced stage cases. For all three IHC markers, positivity was associated with a tendency towards incomplete surgical resection, either R1 or R2. Regarding vascular dissemination, lymph vessel invasion was statistically significantly associated with MUC2 negativity and a propensity for hematogenous dissemination was demonstrated in cases with CDX2 negativity. A higher risk of distant metastasis was exhibited by tumors with MUC2 or MUC5AC loss, but this feature showed no association with the CDX2 pattern (Table 2, Figure 1).

**Figure 2:**
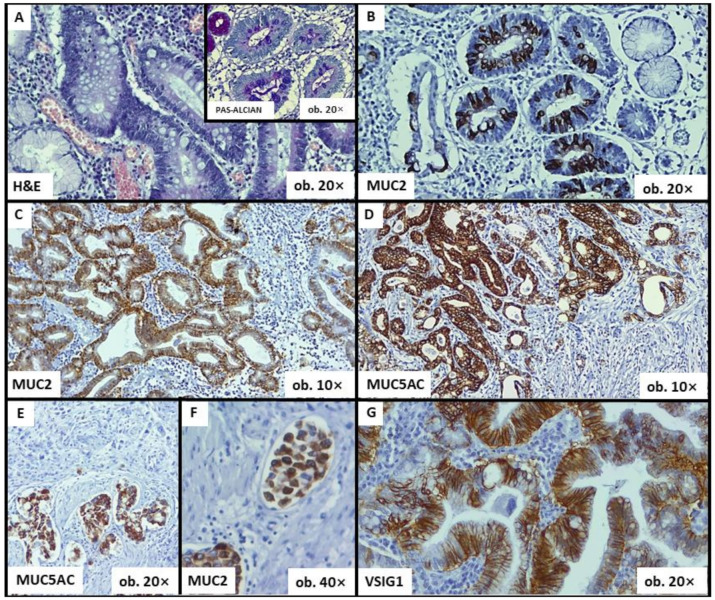
Histological and immunohistochemical features of gastric cancer—The associated-intestinal metaplasia can be diagnosed using hematoxylin–eosin (HE), Alcian blue (**A**) or mucin-related markers (**B**). MUC2 positivity is an indicator of intestinal phenotype (**C**), whereas MUC5AC can highlight the gastric phenotype (**D**). Both MUC5AC and MUC2 can also be seen in areas with perineural invasion (**E**) and within lymph vessel emboli (**F**). VSIG1 shows membrane positivity (**G**).

Although mucins can be seen in areas with perineural invasion (Figure 2), the frequency of occurrence of perineural invasion did not prove to be influenced by MUC2 (*p* = 0.503), MUC5AC (*p* = 0.849) or CDX2 (*p* = 0.465).

**Figure 3:**
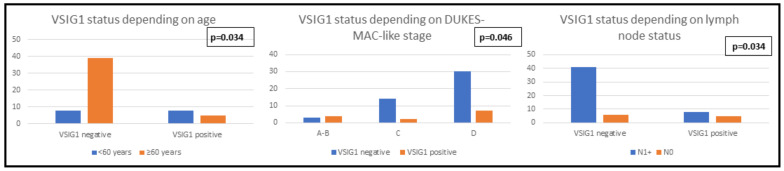
Clinicopathological features of patients with gastric cancer correlated with the immunophenotype of VSIG1.

**Clinicopathological features and immunophenotype of VSIG1:** A statistically significant correlation was demonstrated between VSIG1 expression and Dukes-MAC-like stage: locally advanced cases with lymph node metastases tended to exhibit VSIG1 negativity (Table 3). Although no statistically significant results were obtained for the other clinicopathological features analyzed, an obvious loss of VSIG1 expression can be seen in poorly differentiated tumors which infiltrated the serosa or the perigastric tissues (pT4) but did not show distant metastases (Table 3, Figure 2 and Figure 3).

**Correlation between VSIG1, MUC2, MUC5AC, CDX2 and intestinal metaplasia:** Of the sixty total cases, eleven exhibited MUC2 positivity and seven were VSIG1-negative. A direct association was seen between the immunoexpression of MUC5AC and VSIG1, without any correlation with CDX2 (Table 3).

IM was identified as the precursor lesion in 16 of the 60 cases. Of these, only six were VSIG1 positive but twelve of the sixteen expressed CDX2. MUC5AC, a marker of gastric epithelial differentiation, showed a direct correlation with VSIG1 (Table 3) but did not demonstrate a statistically significant correlation with IM (Table 4).

**Figure 4:**
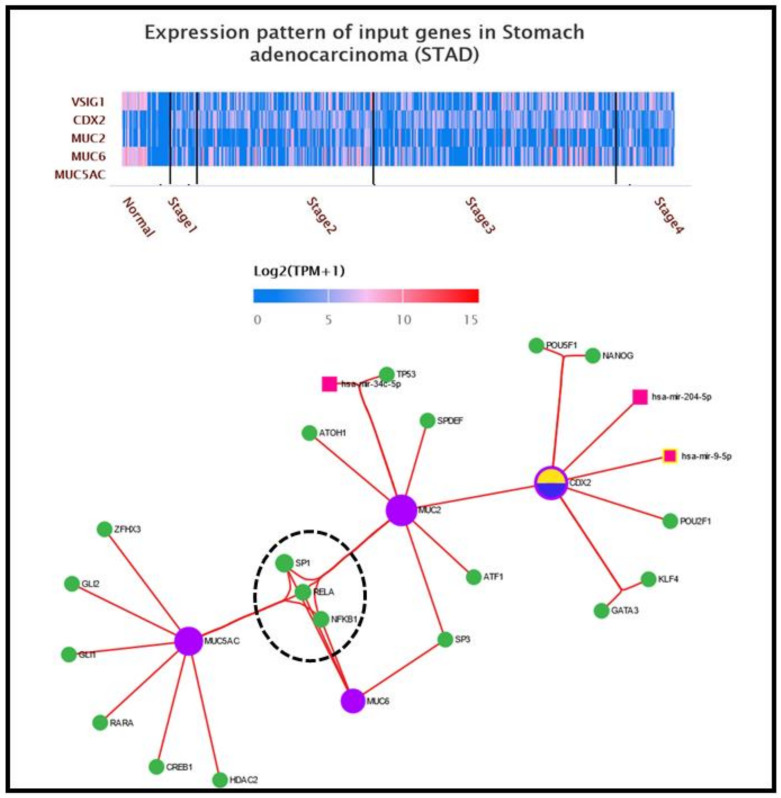
Transcriptional pattern and interaction between molecular pathways of mucins, *CDX2* and *VSIG1.* The heatmap of *MUC2/MUC5AC/CDX2/VSIG1* genes were generated by UALCAN online program using the dataset for gastric carcinoma from TCGA atlas. Integrative molecular network for *MUC2, MUC5AC* and *CDX2* genes in gastric carcinogenesis. Note that of all 19 TFs, only ***SP1, RELA* and *NFKB1*** are connected to all input genes—*CDX2* via *MUC2*, its homologous intestinal phenotype marker.

**Transcriptional signatures related to *MUC2, MUC5AC, CDX2 and VSIG1*:** To validate the obtained data at the protein level, we further examined the molecular pathways of *MUC2*, *MUC5AC*, *CDX2* and *VSIG1* in gastric carcinogenesis, using two different approaches. In the first approach, the expression pattern of the above-mentioned genes was analyzed on a heatmap generated using the UALCAN platform [17]. The expression level of a gene is represented as log_2_(TMP+1), with TMP being transcripts per million. The second approach refers to the construction of a complex molecular network centered by the same target genes, for which the miRNet online platform was used [18]. There were 19 TFs involved in the *MUC2/MUC5AC/CDX2* network: *ZFHX3*, *GLI1*, *GLI2*, *RARA*, *CREB1* and *HDAC2* acting solely on *MUC5AC*; *ATOH1*, *TP53*, *SPDEF* and *ATF1* interacting with *MUC2*; and *POU5F1*, *NANOG*, *POU2F1*, *KLF4* and *GATA3*, which act only on *CDX2*. The remaining four TFs are co-modulators: *SP3* interacts with *MUC2* and *MUC6*, while *SP1*, *RELA* and *NFKB1* regulate all targeted mucin genes. In addition to TFs, three miRNAs are involved in the *MUC2/MUC5AC/CDX2* network: has-mir34c-5p, which is linked to the *MUC2* cascade, has-mir-204-5p and has-mir-9-5p, which act as modulators on the *CDX2* gene. No miRNAs have been demonstrated to act as co-modulators of at least two of the targeted genes. No links have been proven between the mucin-expressing genes/*CDX2* pathway and *VSIG1* gene (Figure 4).

## 3. Discussion

Although several risk factors have been linked to GC, carcinogenesis is mostly a stepwise process that starts with a precursor lesion [19,20]. Heterogeneity is not only a characteristic of GC but it also characterizes the premalignant lesions [21]. The commonest premalignant lesion is the IM, which can be complete or incomplete [22]. Even though IM was first described almost half a century ago, many questions regarding its pathogenesis and relationship to GC remain to be answered. 

To create sub-divisions of GC, MUC2 and MUC5AC have been used to establish the intestinal and gastric tumor phenotypes, respectively [23]. Although *CDX2* is known to be expressed during embryonic development within the gastrointestinal tract mucosa located distally to the duodenum, in GC, CDX2 positivity is also considered an indicator of intestinal phenotype [24]. However, none of these markers are specific to GC. 

Recently, it was hypothesized that a novel molecule, called VSIG1, is a surrogate biomarker for gastric phenotype [25]. VSIG1 has cell–cell adhesion properties and is encoded by the *VSIG1* gene, located on chromosome Xq22.3 [26]. It was first described in 2006 [27]. Although it was initially considered to be restricted to the gastric mucosa, other tissues, both neoplastic and normal, can express VSIG1 [16,28,29]. Of the thirteen published studies related to this relatively recently identified molecule, only three were focused on GC, all of them certifying that most of the adenocarcinomas are marked by VSIG1 and its loss is a negative prognostic factor [15,16,29]. It is not known why some carcinomas are not marked by VSIG1, although gastric mucosa express this protein, or why other tumors display a gastric immunophenotype. 

The current study represents, to the best of our knowledge, the first analysis of possible interactions between VSIG1 and MUC2/CDX2, the surrogate markers of intestinal phenotypes of GC, and between VSIG1 and MUC5AC, a surrogate marker of gastric phenotype. As expected, no correlations between VSIG1 and MUC2 or CDX2 were demonstrated, the only statistically significant association of VSIG1 being with MUC5AC. In our previous research related to VSIG1, we hypothesized that it might be an indicator of the common gastrohepatic lineage of differentiation. For this reason, VSIG1 might be expressed in all tumors with gastric or hepatoid-like features, including hepatocellular carcinoma with gastric phenotype and, conversely, gastric carcinoma with hepatoid phenotype [25,30]. The present study is confirmatory evidence for our theory, emphasizing the correlation with MUC5AC. VSIG1 proved to be, in correlation with MUC5AC, an indicator of GC with gastric but not intestinal phenotypes. This fact is also sustained by VSIG1 negativity in colorectal carcinomas, except those with serrated pathways [16,25,28,30]. 

In daily diagnosis, VSIG1/MUC5AC double positivity might be used as an indicator of gastric origins of unknown metastases. Diagnosis proved to be difficult in some cases, because GCs exhibit variable IHC expression for keratin 7 and 20, two of the most widely used markers for the gastrointestinal tract [31,32]. In such cases, even though not entirely specific for this tumor, VSIG1 and MUC5 were able to confirm the diagnosis. Unfortunately, this tool also has limited utility, emphasizing only CGs with a gastric phenotype.

**Figure 5:**
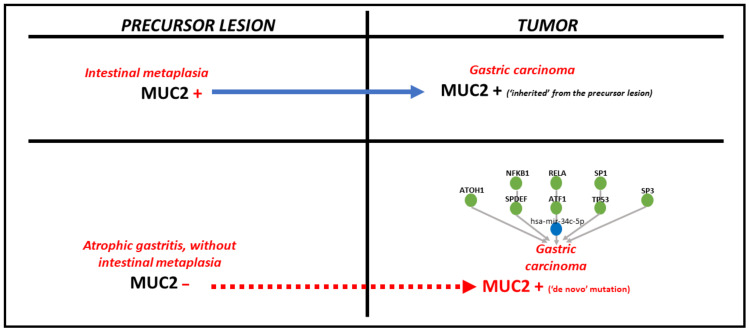
The pathomechanism of MUC2 positivity in gastric cancer, depending on the type of precursor lesion.

Regarding MUC2, a surrogate marker for the intestinal phenotype, it can be hypothesized that, if the premalignant lesion is IM, the tumor cells will inherit a MUC2-positive immunophenotype. Despite this fact, in our study, almost half of MUC2-positive cases were not associated with IM. In this instance, de novo MUC2 positivity might be explained by the interaction with other molecular modulators, such as miRNAs and TFs (Figure 5).

Other studies were focused on de novo positivity for MUC2. This refers to MUC2-positive GCs that are not developed on MUC2-positive precursor lesions. In such cases, another mechanism might be involved in this molecular switch. It might refer to the genetic variability of the *MUC2* gene. It is characterized by the occurrence of a single nucleotide polymorphisms (SNPs)/single nucleotide variations (SNVs) [33].

By using UALCAN and miRNet online platforms [17,18], eight TFs and one miRNA were found to be involved in *MUC2* pathway: *SP1*, *RELA*, *NFKB1*, *ATOH1*, *SPDEF*, *TP53*, *ATF1* and *SP3* and has-mir-34c-5p, respectively (Figure 4 and Figure 5). Of all these, three linkage molecules seem to play role in gastric carcinogenesis, interfering with *MUC5AC: SP1, RELA* and *NFΚB1* (Figure 4). 

*RELA* and *NFΚB1/p50* are TFs of the *NFΚB* family, which includes three additional molecules: *RELB*, *c-REL* and *NFΚB2/p52* [34]. They act on a multitude of genes, modulating their overexpression/upregulation or suppression/downregulation. *MUC2*, an intestinal phenotype mucin gene with no expression in normal gastric mucosa, is upregulated via the *NFΚB* signaling pathway in both gastric IM and gastric carcinoma. *RELA*, one of the most important members of the *NFΚB* family of TFs, is constitutively activated in GC, and it has been demonstrated that tumor progression, especially angiolymphatic dissemination and lymph node and distant metastatic potential, is enhanced through transcriptional regulation and the nuclear translocation of *RELA* [35]. 

Another important ligand within the molecular machinery of mucins is specificity protein 1 (*SP1*), a well-studied TF. The *SP* family of TFs comprises four known molecules, *SP1–4*. However, some authors have suggested that *SP2* should not be considered an *SP* family member, as it is evolutionarily distinct from the other three [36]. *SP1* and *SP3* are major modulators of tumorigenesis in multiple organs, including colorectum, lung, breast and ovary [37,38,39,40]. Although it is yet not completely understood how some members of the *SP* family are involved in gastric carcinogenesis, there is evidence that these molecules act by interacting with not only the MUC family, but also other genes involved in GC, such as *ERBB* family genes [41,42,43]. 

The limitations of the present study, which highlights the possible link between *VSIG1* and *MUC5AC* as an indicator of the development of GC or GC-like tumors with gastric phenotype for the first time in the literature, consist of a few points. Firstly, a low number of cases with high diversity was included. To confirm the geographic-related variability between the two examined groups, we used, as an external control, another study published by our team in 2016, which was focused on the same subject with a significantly larger cohort, in which the ratio between cases with and without intestinal metaplasia was similar to those of the present study [44]. In addition, examination at the protein level only and the lack of comparison between European and non-European patients constitute further limitations. Additionally, *H. pylori* assessment was not performed for all cases and the correlations between *H. pylori* infection and different patterns of expression of the targeted molecules was not possible. As only three previous studies were conducted on VSIG1 expression in GC and other 10 on its expression in other tumors, the study is valuable, and the obtained data need to be confirmed in larger cohorts and experimental studies. 

## 4. Materials and Methods

Case selection: Our study enrolled 60 patients with GC, diagnosed between January 2018 and October 2020 in the Department of Pathology, Clinical County Emergency Hospital, Targu Mures, Romania. The inclusion criteria were surgically treated GC (subtotal or total gastrectomy with D2 or D3 lymphadenectomy) proven to be of epithelial origin, with at least three months postoperative survival and without prior adjuvant therapy intervention. Patients with tumors other than adenocarcinoma or diagnosed solely on biopsy specimens were excluded. The retrospective data collection was approved by the Ethics Committee of Clinical County Emergency Hospital, Targu Mures, Romania (no Ad. 914/04.02.2022).

Tissue microarray (TMA) technique: After the microscopic analysis of all tumor sections stained with hematoxylin and eosin, for each included case, two gastrointestinal pathologists (IJ, SG) re-examined the cases and chose the most representative slide, with no or minimal necrosis or hemorrhage. Cases were sampled from the chosen formalin-fixed paraffin-embedded tissue donor block for each case, using a TMA instrument (Histopathology Ltd., Pécs, Hungary). The extracted tumor tissue was then transferred to a TMA recipient block. To avoid the malposition of the slide during the microscopic assessment, two samples from two different known tissues were placed as controls in the first and last positions of the TMA block.

Histopathology and immunohistochemistry (IHC) analysis: Cases registered in 2020 were staged and graded according to the criteria published in the 5th edition of the World Health Organization (WHO) manual for digestive system tumors. For uniformization, the remaining cases (2018–2019) were reassessed and restaged using the same version of the WHO manual [45]. All cases were also staged according to the newly proposed Dukes-MAC-like staging system [46]. We performed IHC analysis for MUC2, MUC5AC, CDX2 and VSIG1 in all of the 60 primary GCs. The TMA sections (3–5 µm thickness) were deparaffinized and rehydrated, followed by endogenous peroxidase blocking (incubation for 5 min at room temperature with Dako EnVision^TM^ FLEX Peroxidase-Blocking Reagent). Antigen retrieval was performed at high temperature for 30–40 min with a high pH retrieval solution. This step was followed by a 20 min incubation at room temperature with Dako EnVision^TM^ FLEX/HRP detection reagent. EnVision^TM^ FLEX diaminobenzidine was used to develop the stains, and the counterstaining of nuclei was performed with Mayer’s hematoxylin. For each IHC reaction, a 25% cut-off for positivity was established. As an external positive control for MUC5AC, normal gastric mucosa was used, and for MUC2 and CDX2, gastric mucosa with IM was used. In 42 of the 60 cases, H. pylori (polyclonal rabbit, Dako-Agilent, RTU) was also assessed.

Statistical analysis: All included cases were statistically analyzed using the GraphPad Prism (8th edition) platform. For correlations between the clinicopathological features and different patterns of IHC staining for MUC2, MUC5AC, CDX2 and VSIG1, chi-square and Fisher’s exact test were used. A *p*-value under 0.05 and a 95% confidence interval were considered statistically significant. The overall survival rates (OS) were also calculated, with a maximum follow-up period of 43 months.

Gene interaction analysis: We also searched for possible gene interactions, as well as the transcriptional signatures of targeted genes. For this, two online dataset platforms were used, according to the developer’s instructions. Heatmaps for targeted genes were constructed using the UALCAN online platform [17] and a transcription factors (TFs) map was generated using the miRNet online platform [18].

## 5. Conclusions

As well as emphasizing the association with a specific tumor phenotype, the loss of *VSIG1* might indicate a tendency towards lymph node metastases. This molecule could act as a complementary, but not substitutive, tool for *MUC5AC*.

## Figures and Tables

**Table 1:** Clinicopathological parameters of patients with gastric cancer correlated with the presence or absence of tumor-associated intestinal metaplasia.

*Parameter*		*Intestinal Metaplasia (n = 16)*	*Without Metaplasia (n = 44)*	*p-Value*
Sex	Male	31.25% (5)	95.45% (42)	**<0.0001**
	Female	68.75% (11)	4.55% (2)	
Age (years)	<60	62.5% (10)	13.63% (6)	**0.001**
	≥60	37.5% (6)	86.37% (38)	
Histologic Grade	G2	43.75% (7)	45.45% (20)	0.906
	G3	56.25% (9)	54.55% (24)	
Tumor Stage	pT1-2	18.75% (3)	13.63% (6)	0.727
	pT3	31.25% (5)	25% (11)	
	pT4	50% (8)	61.37% (27)	
Dukes-MAC-like Stage	A-B	12.5% (2)	11.36% (5)	0.865
	C	31.25 (5)	25% (11)	
	D	56.25% (9)	63.64% (28)	
Surgical Margins	Positive (R1/R2)	18.75% (3)	20.45% (9)	0.883
	Negative (R0)	81.25% (13)	79.55% (35)	
Lymphatic Invasion	Present (L1)	62.5% (10)	68.18% (30)	0.679
	Absent (L0)	37.5% (6)	31.82% (14)	
Blood Vessel Invasion	Present (V1)	43.75% (7)	54.55% (24)	0.459
	Absent (V0)	56.25% (9)	45.45% (20)	
Lymph Node Metastases	Present (N1+)	87.5% (14)	79.55% (35)	0.481
	Absent (N0)	12.5% (2)	20.45% (9)	
Distant Metastases	Present (M1)	25% (4)	18.18% (8)	0.559
	Absent (M0)	75% (12)	81.82% (36)	

**Table 2:** Clinicopathological parameters of patients with gastric cancer correlated with the immunophenotype of MUC2, MUC5AC and CDX2.

*Parameter*		*MUC2* *Positive* *(n = 11)*	*MUC2* *Negative* *(n = 49)*	*p-Value*	*MUC5AC* *Positive* *(n = 20)*	*MUC5AC* *Negative* *(n = 40)*	*p-Value*	*CDX2* *Positive* *(n = 21)*	*CDX2* *Negative* *(n = 39)*	*p-Value*
Sex	Male	27.27% (3)	89.8% (44)	**<0.0001**	70% (14)	82.5% (33)	0.267	47.62% (10)	94.88% (37)	**0.0002**
	Female	72.73% (8)	10.2% (5)		30% (6)	17.5% (7)		52.38% (11)	5.12% (2)	
Age (years)	<60	36.36% (4)	24.5% (12)	0.42	40% (8)	20% (8)	0.098	33.33% (7)	23.07% (9)	0.391
	≥60	63.64% (7)	75.5% (37)		60% (12)	80% (32)		66.67% (14)	76.93% (30)	
Histologic Grade	G2	45.45% (5)	44.9% (22)	0.973	35% (7)	50% (20)	0.27	71.42% (15)	30.76% (12)	**0.002**
	G3	54.55% (6)	55.1% (27)		65% (13)	50% (20)		28.58% (6)	69.26% (27)	
Tumor Stage	pT1-2	18.18% (2)	14.28% (7)	0.771	15% (3)	15% (6)	**0.011**	33.33% (7)	5.12% (2)	**0.001**
	pT3	18.18% (2)	28.56% (14)		50% (10)	15% (6)		38.09% (8)	20.51% (8)	
	pT4	63.64% (7)	57.16% (28)		35% (7)	70% (28)		28.58% (6)	74.37% (29)	
Dukes-MAC-like Stage	A-B	27.27% (3)	8.16% (4)	0.183	20% (4)	7.5% (3)	0.14	14.28% (3)	10.24% (4)	**0.018**
	C	27.27% (3)	26.54% (13)		35% (7)	22.5% (9)		4.76% (1)	38.46% (15)	
	D	45.45% (5)	65.3% (32)		45% (9)	70% (28)		80.96% (17)	51.3% (20)	
Surgical Margins	R1/2	72.73% (8)	8.16% (4)	**<0.0001**	35% (7)	12.5% (5)	**0.039**	52.38% (11)	2.56% (1)	**<0.0001**
	R0	27.27% (3)	91.84% (45)		65% (13)	87.5% (35)		47.62% (10)	97.44% (38)	
Lymphatic Invasion	L1	27.27% (3)	75.5% (37)	**0.002**	50% (10)	75% (30)	0.052	61.91% (13)	69.24% (27)	0.565
	L0	72.73% (8)	24.5% (12)		50% (10)	25% (10)		38.09% (8)	30.76% (12)	
Blood Vessel Invasion	V1	45.45% (5)	53.06% (26)	0.648	55% (11)	50% (20)	0.714	47.62% (10)	76.92% (30)	**0.021**
	V0	54.55% (6)	46.94% (23)		45% (9)	50% (20)		52.38% (11)	23.08% (9)	
Lymph Node Metastases	N1+	90.9% (10)	79.6% (39)	0.38	85% (17)	80% (32)	0.637	71.42% (15)	81.17% (34)	0.132
	N0	9.1% (1)	20.4% (10)		15% (3)	20% (8)		28.58% (6)	12.83% (5)	
Distant Metastases	M1	63.64% (7)	10.2% (5)	**0.0006**	40% (8)	10% (4)	**0.006**	28.58% (6)	15.38% (6)	0.223
	M0	36.36% (4)	89.8% (44)		60% (12)	90% (36)		71.42% (15)	84.62% (33)	

**Table 3:** Clinicopathological parameters of patients with gastric cancer and mucin-related phenotypes correlated with the immunohistochemical expression of VSIG1.

Parameter		VSIG1 Negative (n = 47)	VSIG1 Positive (n = 13)	*p*-Values
Sex	Male	78.72% (37)	76.92% (10)	0.754
	Female	21.28% (10)	23.08% (3)	
Age (years)	<60	17.02% (8)	61.53% (8)	**0.034**
	≥60	82.98% (39)	38.47% (5)	
Histologic Grade	G2	44.68% (21)	46.15% (6)	0.924
	G3	55.32% (26)	53.85% (7)	
Tumor Stage	pT1-2	10.63% (5)	30.76% (4)	0.165
	pT3	29.78% (14)	15.38% (2)	
	pT4	59.59% (28)	53.85% (7)	
Dukes-MAC-like Stage	A-B	6.38% (3)	30.76% (4)	**0.046**
	C	29.78% (14)	15.38% (2)	
	D	63.82% (30)	53.85% (7)	
Surgical Margins	R1/2	19.14% (9)	23.08% (3)	0.754
	R0	80.86% (38)	76.92% (10)	
Lymphatic Invasion	L1	65.95% (31)	69.24% (9)	0.824
	L0	34.05% (16)	30.76% (4)	
Blood Vessel Invasion	V1	48.93% (23)	61.53% (8)	0.421
	V0	51.07% (24)	38.47% (5)	
Lymph Node Metastases	N1+	87.23% (41)	61.53% (8)	**0.034**
	N0	12.76% (6)	38.47% (5)	
Distant Metastases	M1	19.14% (9)	23.08% (3)	0.754
	M0	80.86% (38)	76.92% (10)	
MUC2	Negative	85.1% (40)	69.24% (9)	0.368
	Positive	14.9% (7)	30.76% (4)	
MUC5AC	Negative	61.7% (29)	84.62% (11)	**0.049**
	Positive	38.3% (18)	15.38% (2)	
CDX2	Negative	76.6% (36)	23.08% (3)	0.405
	Positive	23.4% (11)	76.92% (10)	

**Table 4:** The immunophenotype of gastric cancer cells, in correlation with the presence or absence of tumor-associated intestinal metaplasia.

*Immunohistochemical Marker*		*With Intestinal Metaplasia (n = 16)*	*Without Intestinal Metaplasia (n = 44)*	*p-Values*
VSIG1	Negative	62.5% (10)	84.1% (37)	***0.046***
	Positive	37.5% (6)	15.9% (7)	
MUC2	Negative	62.5% (10)	88.63% (39)	***0.02***
	Positive	37.5% (6)	11.37% (5)	
CDX2	Negative	25% (4)	79.55% (35)	***0.0009***
	Positive	75% (12)	20.45% (9)	
MUC5AC	Negative	68.75% (11)	65.9% (29)	0.836
	Positive	31.25% (5)	34.1% (15)	

## Data Availability

The online data supporting the study results can be found at: http://ualcan.path.uab.edu/analysis.html (accessed on 10 December 2022) and https://www.mirnet.ca/ (accessed on 10 December 2022).
